# Cut throat zone II neck injury and advantage of a feeding jejunostomy

**DOI:** 10.4103/0974-2700.55353

**Published:** 2009

**Authors:** Laleng M Darlong, Neizekhotuo B Shunyu, Rubul Das, Saikat Mallik

**Affiliations:** Department of Surgery, North East Indira Gandhi Regional Institute of Health and Medical Sciences (NEIGRIHMS), Shillong, Meghalaya - 793 018, India; 1 Department of ENT, North East Indira Gandhi Regional Institute of Health and Medical Sciences (NEIGRIHMS), Shillong, Meghalaya - 793 018, India

**Keywords:** Feeding jejunostomy, nerve injuries, zone II neck injury

## Abstract

Penetrating neck injuries account for 5-10% of trauma cases and are potentially life threatening. We report a case of cut- throat zone II neck injury in a 45-year-old male extending up to posterior pharyngeal wall and exposing the underlying cervical vertebra. Tracheostomy was done and wound repair was started from the posterior aspect in layers using 3-0 Vicryl. Intraoperatively, a conscious decision was taken for a feeding jejunostomy for postoperative feeding, which was likely to be prolonged, in view of sensory-nerve damage along the transected pharynx. Prolonged use of Nasogastric tube for postoperative feeding was thus avoided and the discomfort, risk of aspiration and foreign body at injury site eliminated. One week postoperative, the patient experienced severe bouts of coughing and restlessness on oral intake; during this period enteral nutrition was maintained through feeding jejunostomy. At the time of discharge at 1 month, the patient was accepting normal diet orally and was detubated and vocalizing normally. We conclude that postoperative nutrition is an important area to be considered for deep neck wound with nerve injuries due to delayed tolerance to oral feeding till the regeneration of sensory nerves. A feeding jejunostomy or feeding gastrostomy performed simultaneously in such patients with nerve injuries is far superior over nasogastric-tube feeding when prolonged postoperative feeding is expected.

## INTRODUCTION

Penetrating neck injuries which accounts for 5-10% of all trauma cases are potentially dangerous and require emergency treatment. The location of the injury can predict risk and management. Anatomically, the neck can be divided into three major zones for surgery.[1] Zone-1 below cricoids to thoracic inlet, zone-2 from cricoids to the angle of mandible and zone-3 above the angle of mandible. The leading causing of death from penetrating neck injuries is hemorrhage from vascular structures. In a study, by Stone and Callahan,[2] vascular injuries in the neck accounted for 50% of deaths. If there is sign of immediate life-threatening injuries like massive bleeding, expanding hematoma, respiratory distress, hemomediastinum, hemothorax, pneumothorax, pneumomediastinum and hypovolumic shock, then immediate exploration is mandatory. For stable patients, the choice of management remains controversial; either mandatory exploration for all penetrating wounds or selective exploration and observation. Laryngeal mucosal lacerations from penetrating injury should be repaired early preferably within 24 hours. According to Leopold, the time elapsed before repair has an effect on both airway stenosis and voice.[3] For patients undergoing exploration of the neck wound and associated nerve injuries because of disruption of the pharynx, postoperative nutrition is an important area to be considered. Enteral route should always be preferred to parental route, and among the enteral route a nasogastric tube is the one most commonly used because of the ease of insertion intraoperatively. However, a feeding jejunostomy or feeding gastrostomy performed in patients with nerve injuries is far superior to nasogastric tube where early oral feeding gets delayed because of the time required for regeneration of the sensory nerves in the pharynx.

## CASE REPORT

**Figure 1:**
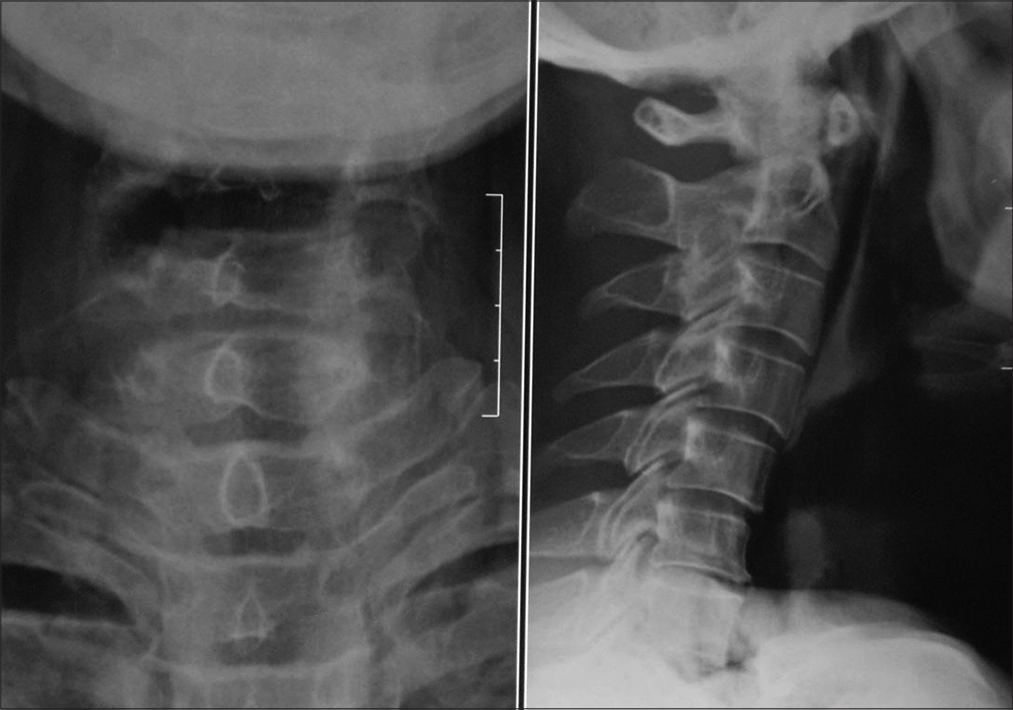
X-ray soft tissue neck showing a transverse air shadow in AP view and below hyoid bone in lateral view at the neck wound

**Figure 2:**
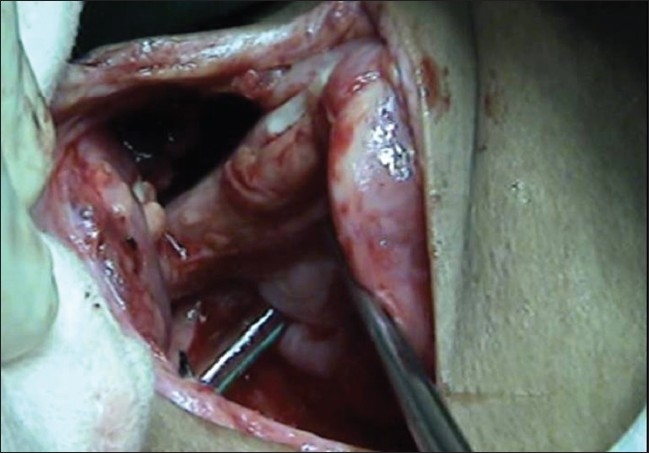
The neck injury exposing the thyroid cartilage and disrupted posterior pharyngeal wall behind the nasogastric tube

**Figure 3:**
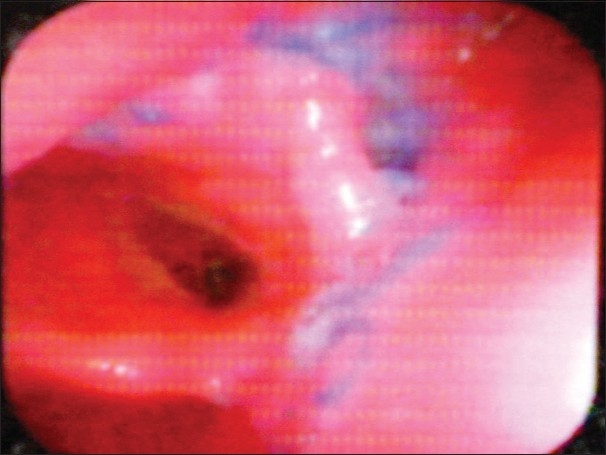
Endoscopic view of the healed wound with sutures *in situ*

**Figure 4:**
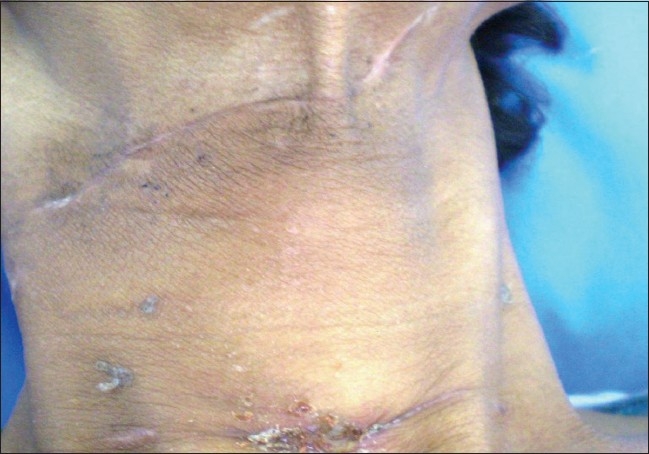
Healed scar of neck injury and tracheostomy site

A 45-year-old male was brought to the casualty 8 hours following an alleged history of cut throat injury. On examination, the patient was in shock and was immediately resuscitated and the blood pressure stabilized at 100/60 mmHg. Local examination of the neck revealed an open deep wound, cut through thyrohyoid membrane. There was profuse frothy salivary discharge from the site and blood oozing from the wound site with no visible arterial bleeding site or surgical emphysema. The wound site was cleared of secretions and airway kept patent. There were no other visible external injuries or neurological deficit. Following this the patient was planned for emergency wound exploration under general anesthesia, and blood grouping and cross-matching done simultaneously along with Hemoglobin and electrolytes. Roentogram was done while in the process of shifting the patient to OT, and chest X-ray was normal with X-ray of soft tissue neck showing the wound area as a transverse air shadow in the AP view and below the hyoid bone in the lateral view [Figure 1]. In the operating table, Intubation was done through the open wound, with the arytenoids as the guide during intubation. After intubation and securing the airway, tracheostomy was done to maintain airway away from the operative site. Detailed on table examination of the wound under general anesthesia confirmed the cut along the thyrohyoid membrane through to the aryepiglottic fold, with the epiglottis totally separated and pulled up; the incised wound was also seen to extend through the posterior pharyngeal wall and exposing the vertebral body [Figure 2]. Using a nasogastric tube inserted across into the esophagus and stomach as a guide, repair of the posterior pharyngeal wall and right side lateral pharyngeal wall was done intraluminally with 3-0 Vicryl. Cut injury through aryepiglottic fold, pharyngoepiglottic fold and salvaged epiglottis were approximated and sutured back extraluminally with 3-0 Vicryl. Second layer suture was given over the first suture. Muscles, fascia and skin were approximated and repaired. Following this an intraoperative decision was taken for a feeding jejunostomy tube in view of the sensory-nerve damage in the transected pharynx and larynx that was likely to interfere with early postoperative oral feeding. We avoided relying on the nasogastric tube for postoperative feeding because of the associated discomfort, risk of aspiration seen with its prolonged use, accidental dislodgement and it itself acting as a foreign body at the site of injury.[4] Before starting orally, we did Upper gastrointestinal endoscopy (UGIE) at 1 week, which revealed no would gap [Figure 3]. Initially at one week postoperatively, the patient experienced severe bouts of cough and restlessness on ingestion of liquids. Following this oral intake was withheld and the patient's nutrition maintained via a feeding jejunostomy, avoiding the discomfort and complications of nasogastric tube. On discharge at 4 weeks, patient was eating and drinking normally without any sign of aspiration and wound completely healed [Figure 4]. He has been detubated and the patient is vocalizing normally with feeding jejunostomy tube removed at 6 weeks.

## DISCUSSION

Neck injuries pose a great challenge because multiple vital structures are vulnerable to injuries in the small, confined unprotected area. This patient had a zone-2 region injury, which is the most frequently involved (60-75%) site for neck injuries. In zone-2, isolated venous and pharyngoesophageal injuries are the most common structures missed clinically during preoperative evaluation. Use of both rigid and flexible esophagoscopy can be complimentary to overcome these problems.[5] Contrast CT scan also aid in the surgical decision making in zone-2 penetrating neck injuries. The debate over merits of mandatory versus selective exploration continues. Meyer and others[6] have reviewed their data and suggested that mandatory exploration is appropriate. Besides these, a very important aspect in the management of above case is the maintenance of nutritional status during the period of recovery where delayed oral feeding is expected in view of the pharyngeal nerve injuries due to the depth of the wound. For postoperative nutrition, the most effective and cheapest is the enteral route. Nasogastric tube for postoperative feeding are to be avoided when it is expected that early oral feeding is unlikely to be tolerated due to sensory-nerve damage in deep wounds extending to the posterior pharyngeal wall. Besides these, the prolong use of a nasogastric tube for feeding during recovery is fraught with patient discomfort, risk of aspiration, accidental dislodgement and also acts as a foreign body at the wound site. [4] The preferred route in such case is feeding jejunostomy or gastrostomy done simultaneously using conventional techniques. Where expertises are available there are several methods of endoscopic placement of feeding tubes, such as percutaneous endoscopic gastrostomy, percutaneous endoscopic gastrostomy with a jejunal tube extension and direct percutaneous endoscopic jejunostomy.[7,8] These endoscopic/percutaneous techniques avoid the laparotomy incision required for placement of conventional feeding tubes. A feeding jejunostomy was preferred over gastrostomy in this case as it was for a benign disease and not for palliative care, and thus preserving the anatomy of the stomach for possible future use as a conduit. This is relevant because of the epidemiologic pattern of certain disease in this region. It has been seen that the prevalence of head and neck cancers in the Northeast India is found to be significantly higher at 54.48% compared to the national average of 30-40%.[9] In a retrospective study in one institution of the Northeast India, esophageal cancers (10.58%) were next only to oropharyngeal cancer.[9] Cancer incidence data generated from six hospital-based cancer registries under National Cancer Control Program (NCRP) have' revealed that in India, Assam has the highest incidence of esophageal cancer, which caters to most areas in the North-east India.[10] Thus bearing this in mind, a feeding jejunostomy is preferred for benign conditions in this region.

## CONCLU	SION

For deep neck injuries, it is very important to keep in mind the sensory nerve injuries of pharynx when injuries extend deep into it. In such cases, postoperative nutrition is very important as early oral feeding is not tolerated till the regeneration of nerves, which usually takes about 4-6 weeks. A feeding jejunostomy/gastrostomy done by conventional or endoscopic technique simultaneously helps in maintaining adequate nutrition during the period of nerve regeneration, unlike the nasogastric tube which itself acts as a foreign body locally with its associated complications.
